# Comparison of different equations for estimating the glomerular filtration rate in pediatric kidney transplant recipients

**DOI:** 10.1007/s00467-025-06942-8

**Published:** 2025-09-22

**Authors:** Paphawadee Sukboonthong, Julaporn Pooliam, Maturin Jantongsree, Achra Sumboonnanonda, Anirut Pattaragarn, Suroj Supavekin, Nuntawan Piyaphanee, Kraisoon Lomjansook, Yarnarin Thunsiribuddhichai, Intraparch Tinnabut, Nuttiporn Khueankong, Thanaporn Chaiyapak

**Affiliations:** 1 Department of Pediatrics, Faculty of Medicine Siriraj Hospital, Mahidol University, Bangkok, 10700 Thailand; 2 Research Department, Faculty of Medicine Siriraj Hospital, Research Development Division, Mahidol University, Bangkok, 10700 Thailand; 3 Research Department, Faculty of Medicine, Siriraj Hospital, Mahidol University, Bangkok, 10700 Thailand; 4 Division of Nephrology, Department of Pediatrics, Faculty of Medicine Siriraj Hospital, Mahidol University, Bangkok, 10700 Thailand; 5 Division of Pediatric Nursing, Department of Nursing, Faculty of Medicine Siriraj Hospital, Mahidol University, Bangkok, 10700 Thailand

**Keywords:** Kidney transplantation, Pediatrics, Estimated glomerular filtration rate, Schwartz–Lyon equation, CKiD under 25, Bedside Schwartz equation

## Abstract

**Background:**

Accurate glomerular filtration rate estimation (eGFR) is essential for managing pediatric kidney transplant recipients. Given the physiology of pediatric patients receiving adult-donor kidneys, identifying the most appropriate plasma creatinine (PCr)-based formula—pediatric or adult-specific—is crucial.

**Methods:**

This cross-sectional study included pediatric kidney transplant recipients (age 1–18 years) who received adult-donor kidneys. We compared agreement thresholds of various pediatric and adult PCr-based GFR equations with CKiD 2012 combined PCr‒cystatin C (PCr-CystC) equation via intraclass correlation coefficients (ICCs), concordance correlation coefficients (CCCs), total deviation index (TDI), P30 performance metric (P30), Bland–Altman plots, and receiver-operating characteristic (ROC) analysis. Correlation between CKiD under 25 (U25) PCr–CystC and reference CKiD 2012 equation was also evaluated.

**Results:**

One hundred twenty samples were collected from 23 recipients (mean age = 14.2 ± 3.4 years) and donors (mean age = 31.7 ± 10.0 years). Schwartz–Lyon equation demonstrated the highest performance with the reference (ICC = 0.913, CCC = 0.911, TDI = 14.0 mL/min/1.73 m^2^, P30 = 99.2%). U25 (ICC = 0.922, CCC = 0.882, P30 = 93.3%), full age spectrum (FAS)-height (ICC = 0.897, CCC = 0.877, P30 = 96.7%), and Bedside Schwartz equations (ICC = 0.850, CCC = 0.819, P30 = 89.2%) showed comparable performance. Bland–Altman plots revealed proportional bias (*p* < 0.05), leading to ROC analysis, which identified eGFR < 70 mL/min/1.73 m^2^ for Schwartz–Lyon, U25, and FAS-height, and < 60 mL/min/1.73 m^2^ for Bedside Schwartz as optimal agreement thresholds, beyond which each equation showed increased bias. Subgroup analyses also showed better performance in patients aged 10–18 years. Additionally, U25 PCr-CystC equation showed excellent agreement with the reference (ICC = 0.993, CCC = 0.990, P30 = 100%).

**Conclusions:**

Schwartz–Lyon equation demonstrated the highest performance among PCr-based equations with the reference in pediatric kidney transplant recipients, particularly when eGFR was < 70 mL/min/1.73 m^2^ and in patients aged 10–18 years. U25 PCr-CystC equation showed best overall agreement with the reference and should be preferred where CystC measurement is feasible.

**Graphical abstract:**

A higher resolution version of the Graphical abstract is available as Supplementary information. 
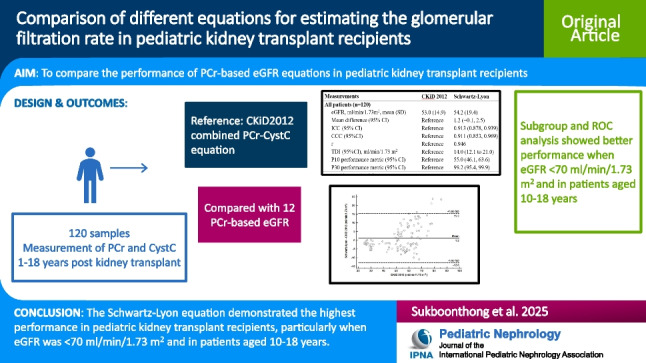

**Supplementary information:**

The online version contains supplementary material available at 10.1007/s00467-025-06942-8.

## Introduction

Compared with chronic dialysis, kidney transplantation is the preferred treatment for pediatric patients with stage 5 chronic kidney disease (CKD), offering superior survival outcomes and quality of life [1, 2]. Accurate estimation of the glomerular filtration rate (eGFR) is essential in managing pediatric kidney transplant recipients, as it can be used to guide medication dosing, monitor allograft rejection, and assess long-term outcomes. Traditionally, GFR measurement using inulin clearance or iohexol plasma clearance has been considered the gold standard. However, these methods are costly, time-consuming, and require specialized laboratory facilities, limiting their widespread use in routine clinical practice [3, 4]. Consequently, endogenous biomarkers such as plasma creatinine (PCr) are commonly used for the eGFR. However, PCr is influenced by muscle mass, diet, and hydration status, often leading to overestimation or underestimation of the GFR in pediatric patients [5].

Although several PCr-based eGFR equations have been developed, most are population-specific. Pediatric-specific equations, such as the Bedside Schwartz, Schwartz–Lyon, and Chronic Kidney Disease in Children (CKiD) under 25 (U25) equations, are commonly used in children, whereas adult-specific equations, including Chronic Kidney Disease Epidemiology Collaboration (CKD-EPI), Modification of Diet in Renal Disease (MDRD), and Cockcroft–Gault, are standard in adults [6]. Recently, full-age spectrum (FAS) equations—such as FAS-age and FAS-height by Pottel et al., the European Kidney Function Consortium (EKFC), and the Lund–Malmö equations—have been introduced to bridge this gap and are applicable across both pediatric and adult populations. However, most of these equations have not been directly validated in pediatric kidney transplant recipients or systematically compared across pediatric and adult age ranges. In pediatric kidney transplant recipients, who typically receive kidneys from adult donors, the optimal equation remains unclear. While some previous pediatric studies have assessed the GFR in pediatric kidney transplant recipients, none have directly compared pediatric- and adult-specific PCr-based eGFR equations [7–10]. Most studies have focused solely on pediatric eGFR equations, leaving a knowledge gap regarding the most appropriate formula for this unique group of patients.

Cystatin C (CystC), a low-molecular–weight protein, is unaffected by muscle mass, sex, and inflammation, making it a promising alternative for eGFR, particularly in pediatric populations above 12 months of age [4, 11–15]. However, it is less accessible and more expensive, limiting its routine use in healthcare centers [16]. The recently updated Kidney Disease: Improving Global Outcomes (KDIGO) 2024 guidelines emphasize the use of the combined PCr-CystC-based eGFR when the PCr-based eGFR alone is less reliable, given its greater accuracy across different populations [17]. A previous study identified the CKiD 2012 combined PCr-CystC-based equation as the most accurate eGFR in pediatric kidney transplant recipients, outperforming both PCr-based and CystC-based equations alone, particularly for measured GFR (mGFR) values below 90 mL/min/1.73 m^2^ [7]. Additionally, another pediatric study showed that the Schwartz combined PCr-CystC-based equation performed better in pediatric kidney transplant recipients than equations based on PCr or CystC alone [8].

Therefore, the aim of this study was to compare the performance of pediatric and adult PCr-based GFR equations against the CKiD 2012 combined PCr-CystC equation in pediatric kidney transplant recipients receiving adult donor kidneys.

## Methods

### Study population

This study included pediatric kidney transplant recipients aged 1–18 years at Siriraj Hospital between April 2024 and November 2024. Eligible patients had undergone kidney transplantation at least one month prior to recruitment, with donors aged over 18 years. The exclusion criteria were as follows: (i) acute kidney injury (AKI), defined as a serum creatinine increase > 0.3 mg/dL within 48 h, > 1.5 times baseline within 7 days, or a urine output < 0.5 mL/kg/h for 6 h [18]; (ii) allograft failure, i.e., requiring peritoneal dialysis or hemodialysis; and (iii) conditions affecting blood urea nitrogen (BUN) levels, including moderate to severe dehydration, gastrointestinal hemorrhage, heart failure, severe infection, liver disease (e.g., cirrhosis, liver failure), use of diuretics, or systemic steroid use > 0.5 mg/kg/day.

### Measurement of Pcr and CystC

PCr was measured using an enzymatic method, and CystC was quantified using a particle-enhanced immunoturbidimetric assay. Both methods were applied throughout the study to ensure reliability. PCr, CystC, and BUN samples were collected simultaneously to maintain standardized procedures.

### Estimation of eGFR

eGFR values were calculated using 12 PCr-based equations: pediatric-specific equations (Bedside Schwartz, Schwartz–Lyon, U25, and Counahan), 4 adult-specific equations (CKD-EPI, MDRD, Cockcroft–Gault, and Baracskay), and 4 full-age spectrum equations (FAS-age, FAS-height, EKFC, and Lund Malmö). These equations were compared with the CKiD 2012 combined PCr-CystC equation. The equations used were as follows:

(i) Bedside Schwartz [19]: eGFR = *K* × height (cm)/PCr, where *K* = 0.413
(ii) Schwartz‒Lyon [20]: eGFR = *K* × height (cm)/PCr, where *K* = 0.413 for boys > 13 years and *K* = 0.367 for others
(iii) U25 [21] = *K* × height (m)/PCr, where *K* is calculated as the following:
  Male, *K* is calculated as age 1 to < 12 years old = 39.0 × 1.008^(age−12)^, age 12 to < 18 years old = 39.0 × 1.045^(age−12)^, and age 18–25 years old = 50.8
   Female, *K* is calculated as age 1 to < 12 years old = 36.1 × 1.008^(age−12^), age 12 to < 18 years old = 36.1 × 1.023^(age−12)^, and age 18–25 years old = 41.4
(iv) CKD-EPI [22]: eGFR = 142 × (PCr/*A*)^B^ × 0.9938.^age^ × (1.012 if female)
  Female: *A* = 0.7, *B* =  − 0.241 if PCr ≤ 0.7; *A* = 0.7, B =  − 1.2 if PCr > 0.7
  Male: *A* = 0.9, *B* =  − 0.302 if PCr ≤ 0.9; *A* = 0.9, B =  − 1.2 if PCr ≥ 0.9
(v) MDRD [23]: eGFR = 175 × PCr^−1.154^ × age.^−0.203^ × 1.212 (if the patient is black) × 0.742 (if female)
(vi) Cockcroft-Gault [6]: eGFR = (140-age) × (weight, kg) × (0.85 if female)/(72 × PCr)
(vii) FAS-age [24]: eGFR = 107.3/(PCr/*Q*), with *Q* matching age
(viii) FAS-height [24]: eGFR = 107.3/(PCr/*Q*), with *Q* matching height
(ix) EKFC [25]: eGFR = 107.3 × (PCr/*Q*)^−0.322^ if PCr/*Q* < 1 or eGFR = 107.3 × (PCr/*Q*)^−1.132^ if PCr/*Q* ≥ 1, with *Q* values correspond to mean PCr for age and sex specific populations
(x) Lund-Malmö [26]: PCr (µmol/L) = PCr (mg/dL) × 88.4
  Female: If PCr < 150 µmol/L, eGFR = exp (2.5 + 0.0121 × (150 − PCr) − 0.0158 × age + 0.438 × ln (age))
    If PCr ≥ 150 µmol/L, eGFR = exp (2.5–0.926 × ln (PCr) − 0.0158 × age + 0.438 × In (age))
  Male: If PCr < 180 µmol/L, eGFR = exp (2.56 + 0.00968 × (180 − PCr) − 0.0158 × age + 0.438 × ln (age))
    If PCr ≥ 180 µmol/L, eGFR = exp (2.56 − 0.926 × ln (PCr) − 0.0158 × age + 0.438 × ln (age))
(xi) Counahan [6]: eGFR = 0.43 × height/PCr
(xii) Baracskay [27]: eGFR = 1/2 (100/PCr) + 88 − age
(xiii) CKiD 2012 combined PCr-CystC [13]: eGFR = 39.8 × (height/PCr)^0.456^ × (1.8/CystC)^0.418^ × (30/BUN)^0.079^ × (1.076^male^) (1.00^female^) × (height/1.4).^0.179^
(xiv) U25 combined PCr-CystC [21]: eGFR = (PCr-based U25 eGFR + CystC-based U25 eGFR)/2
  CystC-based U25 eGFR = *K* × (1/CystC), where *K* is calculated as the following:
    Male, *K* is calculated as age 1 to < 15 years old = 87.2 × 1.011(age−15), age 15 to < 18 years old = 87.2 × 0.96(age−15), and age 18 to 25 years old = 77.1
    Female, *K* is calculated as age 1 to < 12 years old = 79.9 × 1.004(age−12), age 12 to < 18 years old = 79.9 × 0.974(age−12), and age 18–25 years old = 68.3

### Statistical analysis

Descriptive statistics were used to summarize patient demographics. Continuous data were presented as the means with standard deviations (SDs) for normally distributed variables or medians with interquartile ranges (IQRs) for non-normally distributed variables. Categorical data are expressed as numbers and percentages. Mean differences in eGFR were analyzed using paired Student’s *t* tests for normally distributed data or Mann‒Whitney *U* tests for nonnormally distributed data. A *p* value < 0.05 was considered to indicate statistical significance.

The performance of the 12 PCr-based eGFR equations was assessed against the CKiD 2012 combined PCr-CystC equation using intraclass correlation coefficients (ICCs), concordance correlation coefficient (CCC), total deviation index (TDI), 10% of the reference (P10 performance metric, P10), and 30% of the reference (P30 performance metric, P30) [17]. The ICC values were interpreted as follows [28]: > 0.9 (excellent), 0.75–0.9 (good), 0.5–0.75 (moderate), and < 0.5 (poor). The CCC values were interpreted as follows [29]: > 0.99 (almost perfect), 0.95–0.99 (substantial), 0.90–0.95 (moderate), < 0.9 (poor). Pearson’s correlation coefficients (*r*) were interpreted as follows [30]: 0.90–1.00 (very strong correlation), 0.70–0.89 (strong correlation), 0.40–0.69 (moderate correlation), 0.10–0.39 (weak correlation), and 0.00–0.10 (negligible correlation). P10 and P30 performance metric represent the proportions of eGFR estimates falling within 10% and 30%, respectively, of the reference eGFR; higher values of P10 and P30 performance metric indicate greater performance. Agreement between the PCr-based eGFR equation and the CKiD 2012 combined PCr-CystC equation was evaluated via Bland‒Altman plots, which calculate bias as the mean difference between equations. Proportional bias was assessed using linear regression analysis of Bland–Altman plots, and if significant bias was detected (*p* < 0.05), receiver-operating characteristic (ROC) curves were used to determine the eGFR thresholds beyond which the agreement between methods deteriorated.

Statistical analyses were conducted using MedCalc version 19.6.4 (MedCalc Software Ltd., Ostend, Belgium) for Bland‒Altman plots, CCC, and TDI and IBM® SPSS® Statistics version 29.0 (IBM Corp, Armonk, NY, USA) for all other analyses.

## Results

### Demographic and clinical characteristics

Twenty-three pediatric kidney transplant recipients aged 1–18 years at Siriraj Hospital were included in the study. All met the inclusion criteria, resulting in 120 eGFR measurements. Most patients underwent multiple assessments during different blood work periods, with a median of 4.0 (4.0) GFR measurements per patient.

The baseline demographic and clinical characteristics are summarized in Table 1. The mean age was 14.2 years (3.4), with a mean body weight of 40.2 kg (16.3) and a mean height of 142.5 cm (19.9). The main causes of CKD were glomerular diseases (56.5%) and congenital anomalies of the kidney and urinary tract (30.4%). All patients received their first kidney transplant, 52.2% from cadaveric donors, with a mean age of 32.1 years (10.6). Immunosuppressive therapies received were prednisolone and tacrolimus for all patients, supplemented with either mycophenolate mofetil (74.0%) or everolimus (26.0%). Of the 23 patients included, 17 (73.9%) demonstrated stable graft function at 6 months post-transplantation. Among the six remaining patients, the underlying conditions were antibody-mediated rejection (ABMR, *n* = 2), recurrent IgA nephropathy (*n* = 1), recurrent focal segmental glomerulosclerosis (FSGS, *n* = 1), and CMV infection requiring ganciclovir treatment (*n* = 2). Importantly, despite these events, all six patients had stable graft function without acute kidney injury (AKI) at the time of study enrollment.

**Table 1:** Demographic and clinical characteristics of 23 pediatric transplant recipients

Characteristics	Number (*n* = 23)
Male, *n* (%)	13 (56.5)
Age, years (SD)	14.2 (3.4)
Cause of CKD, *n* (%)	
Glomerular disease	13 (56.5)
CAKUT	7 (30.4)
Unknown	1 (4.3)
Others	2 (8.7)
Weight, kg (SD)	40.2 (16.3)
Height, cm (SD)	142.5 (19.9)
Cadaveric donor, *n* (%)	12 (52.2)
Donor age, years (SD)	32.1 (10.6)
Native nephrectomy, *n* (%)	2 (8.7)
First kidney transplant, *n* (%)	23 (100)
Creatinine, mg/dL (SD)	1.2 (0.6)
Cystatin C, mg/dL	1.8 (0.6)
Immunosuppressive drugs, *n* (%)	
Prednisolone	23 (100)
Tacrolimus	23 (100)
Mycophenolate mofetil	17 (74)
Everolimus	6 (26)

*CAKUT* congenital anomalies of the kidney and urinary tract, *CKD* chronic kidney disease, *SD* standard deviation

### Formula performance of 12 Pcr-based eGFR equations compared with the CKiD 2012 combined Pcr-CystC equation

The performance of the 12 PCr-based eGFR equations compared with the CKiD 2012 combined PCr-CystC equation is summarized in Tables 2 and 3. A total of 12 PCr-based eGFR equations were compared against the CKiD 2012 combined equation. Most equations overestimated the eGFR compared with the reference, with the smallest bias observed for Schwartz–Lyon (mean difference, 1.2 mL/min/1.73 m^2^; 95% CI, − 0.1, 2.5) and the largest for Baracskay (mean difference, 72.0; 95% CI, 68.7, 75.3). The Schwartz–Lyon equation showed the highest agreement with the reference (ICC = 0.913, CCC = 0.911, TDI = 14.0 mL/min/1.73 m^2^, P30 = 99.2%). The U25 (ICC = 0.922, CCC = 0.882, TDI = 20.0 mL/min/1.73 m^2^, P30 = 93.3%), FAS–height (ICC = 0.897, CCC = 0.877, TDI = 18.5 mL/min/1.73 m^2^, P30 = 96.7%), and Bedside Schwartz (ICC = 0.850, CCC = 0.819, TDI = 23.8 mL/min/1.73 m^2^, P30 = 89.2%) equations showed comparable performance. In contrast, MDRD and Baracskay exhibited poor concordance (CCC = 0.212 and 0.099, respectively) and low P30 performance metric (P30 = 26.7 and 0, respectively). Subgroup analyses by age and eGFR strata consistently supported the superior performance of the Schwartz–Lyon equation, particularly in patients aged 10–18 years and those with GFR < 75 mL/min/1.73 m^2^. In addition, the U25, FAS–height, and Bedside Schwartz equations showed comparable performance and also performed well in this subgroup.

**Table 2:** Correlation and performance metric of the creatinine-based eGFR equations compared with the CKiD 2012 combined plasma creatinine–cystatin C equation (*n* = 120)

Measurements	CKiD 2012	Bedside Schwartz	Schwartz-Lyon	U25	CKD-EPI	MDRD	Cockcroft-Gault
**All patients** (***n*** **= 120)**							
eGFR, mL/min/1.73 m^2^, mean (SD)	53.0 (14.9)	58.3 (22.9)	54.2 (19.4)	58.3 (19.8)	66.6 (24.4)	97.2 (60.8)	62.4 (26.5)
Mean difference (95% CI)	Reference	5.3 (3.4, 7.2)	1.2 (− 0.1, 2.5)	5.3 (4.0, 6.5)	13.6 (11.2, 16.0)	44.2 (34.8, 53.5)	9.4 (6.4, 12.5)
ICC (95% CI)	Reference	0.850 (0.791, 0.893)	0.913 (0.878, 0.939)	0.922 (0.890, 0.945)	0.786 (0.70, 0.846)	0.312 (0.148, 0.470)	0.694 (0.588, 0.776)
CCC (95%CI)	Reference	0.819 (0.753, 0.885)	0.911 (0.853, 0.969)	0.882 (0.831, 0.932)	0.641 (0.557, 0.724)	0.212 (0.082, 0.342)	0.632 (0.528, 0.737)
* r*	Reference	0.930	0.946	0.955	0.884	0.689	0.812
TDI (95%CI), mL/min/1.73 m^2^	Reference	23.8 (20.2 to 33.4)	14.0 (12.1 to 21.0)	20.0 (16.2–24.0)	37.1 (30.2 to 43.2)	172.6 (92.8, 243.1)	45.4 (31.0, 54.5)
P10 performance metric (95% CI)	Reference	40.0 (31.7, 48.9)	55.0 (46.1, 63.6)	67.5 (58.7, 75.2)	26.7 (19.6, 35.2)	3.3 (1.3, 8.2)	20.0 (13.8, 28.0)
P30 performance metric (95% CI)	Reference	89.2 (82.3, 93.6)	99.2 (95.4, 99.9)	93.3 (87.4, 96.6)	54.2 (45.3, 62.8)	26.7 (19.6, 35.2)	60.8 (51.9, 69.1)
**Age < 10 years** (***n*** **= 26)**							
eGFR, mL/min/1.73 m^2^, mean (SD)	64.6 (8.0)	83.2 (15.6)	73.9 (13.9)	73.0 (15.0)	74.4 (11.1)	185.2 (70.3)	67.4 (17.7)
Mean difference (95% CI)	Reference	18.6 (8.5)	9.3 (6.9)	8.3 (8.4)	9.8 (12.6)	120.6 (64.3)	2.8 (15.6)
ICC (95% CI)	Reference	0.765 (0.542, 0.887)	0.816 (0.631, 0.913)	0.759 (0.531, 0.884)	0.149 (0.246, 0.501)	0.174 (− 0.221, 0.520)	0.351 (− 0.035, 0.645)
CCC (95%CI)	Reference	0.360 (− 0.031, 0.656)	0.609 (0.291, 0.806)	0.611 (0.294, 0.808)	0.098 (− 0.300, 0.468)	0.045 (− 0.349, 0.425)	0.344 (− 0.050, 0.645)
* r*	Reference	0.943	0.943	0.914	0.157	0.775	0.467
TDI (95%CI), mL/min/1.73 m^2^	Reference	35.9 (24.6, 37.1)	22.8 (14.0, 23.8)	25.4 (15.6, 26.2)	28.3 (25.2, 30.2)	249.2 (196.3, 264.0)	32.1 (22.2, 34.0)
P10 performance metric (95% CI)	Reference	7.7 (2.1, 24.1)	38.5 (22.4, 57.5)	46.2 (28.8, 64.5)	53.8 (35.4, 71.3)	0 (0, 12.9)	26.9 (13.7, 46.1)
P30 performance metric (95% CI)	Reference	57.7 (39.0, 74.5)	96.2 (81.1, 99.3)	92.3 (75.9, 97.9)	65.4 (46.2, 80.6)	0 (0, 12.9)	80.8 (62.1, 91.5)
**Age 10–15 years** (***n*** **= 38)**							
eGFR, mL/min/1.73 m^2^, mean (SD)	44.3 (16.6)	48.0 (23.9)	44.5 (19.9)	46.5 (20.0)	53.8 (28.9)	72.1 (29.2)	47.8 (30.2)
Mean difference (95% CI)	Reference	3.7 (1.3)	0.13 (4.7)	2.1 (4.6)	9.5 (13.0)	27.8 (14.5)	3.5 (15.7)
ICC (95% CI)	Reference	0.920 (0.852, 0.958)	0.967 (0.937, 0.983)	0.969 (0.942, 0.984)	0.848 (0.726, 0.918)	0.813 (0.668, 0.898)	0.792 (0.635, 0.886)
CCC (95%CI)	Reference	0.906 (0.825, 0.950)	0.967 (0.937, 0.983)	0.963 (0.929, 0.981)	0.785 (0.621, 0.883)	0.482 (0.192, 0.695)	0.784 (0.620, 0.883)
* r*	Reference	0.983	0.983	0.986	0.983	0.946	0.939
TDI (95%CI), mL/min/1.73 m^2^	Reference	20.2 (12.6, 23.1)	9.4 (6.1 to 12.9)	11.7 (6.3, 15.3)	31.4 (23.8, 34.4)	49.5 (43.0, 72.0)	41.9 (14.6, 45.3)
P10 performance metric (95% CI)	Reference	47.4 (32.5, 62.7)	65.8 (49.9, 78.8)	78.9 (63.7, 88.9)	26.3 (15.0, 42.1)	0 (0, 9.2)	18.4 (9.2, 33.4)
P30 performance metric (95% CI)	Reference	94.7 (82.7, 98.5)	100.0 (90.8, 100.0)	100.0 (90.8, 100.0)	65.8 (49.9, 78.8)	2.6 (0.5, 13.5)	57.9 (42.2, 72.2)
**Age > 15 years** (***n*** **= 56)**							
eGFR, mL/min/1.73 m^2^, mean (SD)	53.5 (12.2)	53.7 (15.7)	51.7 (14.2)	59.5 (16.6)	71.7 (22.3)	73.3 (22.3)	70.1 (23.3)
Mean difference (95% CI)	Reference	0.2 (7.2)	− 1.8 (5.9)	6.0 (6.8)	18.2 (12.3)	19.8 (14.3)	16.6 (15.4)
ICC (95% CI)	Reference	0.870 (0.788, 0.922)	0.902 (0.838, 0.941)	0.891 (0.821, 0.935)	0.767 (0.633, 0.857)	0.681 (0.511, 0.800)	0.657 (0.479, 0.784)
CCC (95%CI)	Reference	0.870 (0.787, 0.922)	0.894 (0.825, 0.937)	0.821 (0.712, 0.892)	0.507 (0.281 to 0.679)	0.423 (0.180, 0.617)	0.470 (0.237, 0.653)
* r*	Reference	0.898	0.913	0.934	0.911	0.809	0.801
TDI (95%CI), mL/min/1.73 m^2^	Reference	14.0 (10.5, 15.9)	11.7 (8.3, 14.0)	20.0 (16.2, 24.1)	42.2 (33.8, 44.0)	54.7 (32.8, 60.9)	51.3 (27.8, 55.6)
P10 performance metric (95% CI)	Reference	50.0 (37.3, 62.7)	55.4 (42.4, 67.6)	69.5 (56.7, 80.1)	14.3 (74.2, 25.7)	7.1 (2.8, 17.0)	17.9 (10.0, 29.8)
P30 performance metric (95% CI)	Reference	100.0 (93.6, 100.0)	100.0 (93.6, 100.0)	89.3 (78.5, 95.0)	41.1 (29.2, 54.1)	55.4 (42.4, 67.6)	53.6 (40.7, 66.0)
**CKiD eGFR < 75 mL/min/1.73 m**^**2**^ (***n*** **= 114)**							
eGFR, mL/min/1.73 m^2^, mean (SD)	51.7 (14.0)	55.8 (20.4)	52.1 (17.4)	56.2 (17.9)	65.4 (24.0)	90.3 (48.4)	61.1 (26.1)
Mean difference (95% CI)	Reference	4.1 (9.3)	0.5 (6.3)	4.6 (6.2)	13.7 (12.8)	38.7 (40.3)	9.4 (16.5)
ICC (95% CI)	Reference	0.859 (0.802, 0.900)	0.919 (9.885, 0.944)	0.927 (0.896, 0.949)	0.788 (0.707, 0.849)	0.360 (0.189, 0.510)	0.688 (0.577, 0.773)
CCC (95%CI)	Reference	0.835 (0.770, 0.883)	0.919 (0.884, 0.943)	0.891 (0.846, 0.924)	0.634 (0.510, 0.733)	0.226 (0.044, 0.394)	0.625 (0.498, 0.725)
* r*	Reference	0.921	0.941	0.956	0.907	0.675	0.825
TDI (95%CI), mL/min/1.73 m^2^	Reference	22.0 (17.2, 24.2)	13.2 (11.1, 14.4)	17.7 (15.5, 20.2)	36.9 (30.2, 41.9)	96.1 (88.5, 197.2)	43.7 (29.9, 50.3)
P10 performance metric (95% CI)	Reference	42.1 (33.0, 51.2)	57.0 (47.8, 65.7)	71.1 (62.2, 78.6)	26.3 (19.1, 35.1)	3.5 (1.4, 8.7)	16.7 (10.9, 24.6)
P30 performance metric (95% CI)	Reference	91.2 (84.6, 95.2)	100.0 (96.7, 100.0)	94.7 (89.0, 97.6)	52.6 (43.5, 61.6)	28.1 (20.7, 36.9)	59.6 (50.5, 68.2)
**CKiD eGFR ≥ 75 mL/min/1.73 m**^**2**^ (***n*** **= 6)**							
eGFR, mL/min/1.73 m^2^, mean (SD)	78.6 (3.5)	105.8 (12.4)	94.0 (11.0)	97.6 (9.6)	90.3 (18.3)	227.3 (116.7)	88.8 (21.7)
Mean difference (95% CI)	Reference	27.2 (9.5)	15.4 (8.2)	19.0 (6.9)	11.7 (20.7)	148.7 (114.3)	10.2 (23.5)
ICC (95% CI)	Reference	0.444 (− 0.467, 0.898)	0.489 (− 0.420, 0.908)	0.539 (− 0.363, 0.920)	− 0.235 (− 0.840, 0.632)	0.041 (− 0.736, 0.772)	− 0.141 (− 0.810, 0.686)
CCC (95%CI)	Reference	0.081 (− 0.782, 0.838)	0.176 (− 0.742, 0.864)	0.121 (− 0.766, 0.849)	− 0.168 (− 0.862, 0.745)	0.016 (− 0.806, 0.817)	− 0.117 (− 0.848, 0.768)
* r*	Reference	0.837	0.837	0.828	− 0.629	0.679	− 0.444
TDI (95%CI), mL/min/1.73 m^2^	Reference	37.0 (30.2, 37.1)	23.7 (18.2, 23.8)	26.2 (21.1, 26.2)	38.4 (18.2, 43.9)	260.8 (194.6, 264.0)	44.5 (4.4, 57.6)
P10 performance metric (95% CI)	Reference	0 (0, 39.0)	16.7 (3.0, 56.4)	0 (0, 39.0)	33.3 (9.7, 70.0)	0 (0, 39.0)	83.3 (43.7, 97.0)
P30 performance metric (95% CI)	Reference	50.0 (18.8, 81.2)	83.3 (43.7, 97.0)	66.7 (30.0, 90.3)	83.3 (43.7, 97.0)	0 (0, 39.0)	83.3 (43.7, 97.0)

*95% CI* 95% confidence interval, *eGFR* estimated glomerular filtration rate, *CKiD* Chronic Kidney Disease in Children, *U25* the CKiD under 25, *CKD-EPI* Chronic Kidney Disease Epidemiology Collaboration, *MDRD* Modification of Diet in Renal Disease, *ICC* intraclass correlation coefficient, *CCC* concordance correlation coefficient, *r* Pearson’s correlation coefficient, *TDI* total deviation index, *SD* standard deviation, *P10 performance metric* proportion within 10% of the reference, *P30 performance metric* proportion within 30% of the reference

**Table 3:** Correlation and accuracy of the creatinine-based eGFR equations compared with the CKiD 2012 combined plasma creatinine–cystatin C equation (*n* = 120)

Measurements	CKiD 2012	FAS-age	FAS-height	EKFC	Lund_malmö	Counahan	Baracskay
**All patients** (***n*** **= 120)**							
eGFR, mL/min/1.73 m^2^, mean (SD)	53.0 (14.9)	68.6 (21.5)	56.9 (21.4)	63.8 (21.7)	67.2 (22.5)	60.7 (23.8)	125.0 (26.8)
Mean difference (95% CI)	Reference	15.6 (9.4)	3.9 (8.4)	10.8 (9.8)	14.2 (10/6)	7.7 (5.6, 9.7)	72.0 (68.7, 75.3)
ICC (95% CI)	Reference	0.869 (0.818, 0.907)	0.897 (0.855, 0.927)	0.861 (0.807, 0.901)	0.846 (0.786, 0.890)	0.836 (0.773, 0.883)	0.644 (0.526, 0.738)
CCC (95% CI)	Reference	0.641 (0.575, 0.707)	0.877 (0.825, 0.929)	0.738 (0.669, 0.806)	0.663 (0.593, 0.732)	0.777 (0.712, 0.843)	0.099 (0, 0.215)
* r*	Reference	0.929	0.956	0.924	0.920	0.930	0.759
TDI (95%CI), mL/min/1.73 m^2^	Reference	33.3 (29.5, 37.3)	18.5 (15.2, 24.4)	29.7 (25.2 to 34.3)	31.1 (30.5, 33.5)	27.6 (23.9, 38.0)	107.6 (100.9, 122.2)
P10 performance metric (95% CI)	Reference	10.8 (6.4, 17.7)	36.7 (28.6, 45.6)	31.7 (24.0, 40.5)	14.2 (9.0, 21.5)	44.2 (35.6, 53.1)	0 (0, 0)
P30 performance metric (95% CI)	Reference	51.7 (42.9, 60.4)	96.7 (91.7, 98.7)	76.7 (68.3, 83.3)	61.7 (52.7, 69.9)	80.0 (72.0, 86.2)	0 (0, 0)
**Age < 10 years** (***n*** **= 26)**							
eGFR, mL/min/1.73 m^2^, mean (SD)	64.6 (8.0)	84.5 (14.3)	77.0 (14.4)	81.6 (13.9)	91.6 (9.3)	86.5 (16.3)	165.5 (18.4)
Mean difference (95% CI)	Reference	0.927	0.940	0.919	0.890	21.9 (9.2)	100.9 (11.8)
ICC (95% CI)	Reference	0.791 (0.586, 0.900)	0.798 (0.600, 0.904)	0.794 (0.592, 0.902)	0.880 (0.751, 0.944)	0.746 (0.511, 0.878)	0.653 (0.362, 0.828)
CCC (95% CI)	Reference	0.319 (–0.078, 0.629)	0.510 (0.153, 0.749)	0.375 (− 0.014, 0.666)	0.151 (− 0.251, 0.509)	0.304 (− 0.095, 0.618)	0.025 (− 0.366, 0.408)
* r*	Reference	0.898	0.925	0.883	0.891	0.944	0.891
TDI (95%CI), mL/min/1.73 m^2^	Reference	32.6 (24.2, 34.0)	26.8 (17.5, 27.9)	28.7 (22.1, 29.9)	33.3 (30.9, 34.6)	40.6 (28.3, 42.1)	123.1 (111.5, 124.1)
P10 performance metric (95% CI)	Reference	3.8 (0.7, 18.9)	15.4 (6.2, 33.5)	3.8 (0.7, 18.9)	0 (0, 12.9)	0 (0, 12.9)	0 (0, 12.9)
P30 performance metric (95% CI)	Reference	34.6 (19.4, 53.8)	88.5 (71.0, 96.0)	61.5 (42.5, 77.6)	7.7 (2.1, 24.1)	38.5 (22.4, 57.5)	0 (0, 12.9)
**Age 10–15 years** (***n*** **= 38)**							
eGFR, mL/min/1.73 m^2^, mean (SD)	44.3 (16.6)	57.9 (22.1)	46.5 (23.5)	53.6 (22.6)	56.3 (22.4)	50.1 (24.9)	115.5 (19.4)
Mean difference (95% CI)	Reference	13.5 (9.0)	2.1 (7.8)	9.3 (9.2)	11.9 (8.2)	5.7 (9.1)	71.1 (7.3)
ICC (95% CI)	Reference	0.895 (0.807, 0.944)	0.927 (0.865, 0.962)	0.892 (0.803, 0.943)	0.914 (0.840, 0.954)	0.907 (0.829, 0.951)	0.919 (0.851, 0.957)
CCC (95%CI)	Reference	0.722 (0.523–0.846)	0.922 (0.855, 0.959)	0.804 (0.651, 0.894)	0.772 (0.601, 0.876)	0.875 (0.771, 0.934)	0.105 (–0.222, 0.411)
* r*	Reference	0.932	0.985	0.935	0.955	0.983	0.931
TDI (95%CI), mL/min/1.73 m^2^	Reference	31.7 (20.0, 43.2)	18.5 (10.9, 21.6)	27.4 (15.1, 41.2)	30.3 (17.0, 36.4)	24.2 (15.6 to 26.9)	87.0 (75.2, 94.7)
P10 performance metric (95% CI)	Reference	15.8 (7.4, 30.4)	31.6 (19.1, 47.5)	23.7 (13.0, 39.2)	10.5 (4.2, 24.1)	57.9 (42.2, 72.2)	0 (0, 9.2)
P30 performance metric (95% CI)	Reference	42.1 (27.9, 57.8)	97.4 (86.5, 99.5)	84.2 (69.6, 92.6)	73.7 (58.0, 85.0)	84.2 (69.6, 92.6)	0 (0, 9.2)
**Age > 15 years** (***n*** **= 56)**							
eGFR, mL/min/1.73 m^2^, mean (SD)	53.5 (12.2)	68.5 (19.4)	54.6 (15.8)	62.4 (19.2)	63.3 (18.5)	55.9 (16.4)	112.6 (12.2)
Mean difference (95% CI)	Reference	15.0 (10.0)	1.2 (6.5)	8.9 (10.2)	9.9 (9.5)	2.4 (7.6)	59.1 (7.7)
ICC (95% CI)	Reference	0.808 (0.693, 0.882)	0.895 (0.827, 0.937)	0.799 (0.680, 0.877)	0.817 (0.707, 0.880)	0.861 (0.773, 0.916)	0.800 (0.681, 0.878)
CCC (95%CI)	Reference	0.565 (0.355, 0.721)	0.892 (0.821, 0.935)	0.693 (0.526, 0.809)	0.683 (0.512, 0.802)	0.849 (0.754, 0.909)	0.063 (− 0.204, 0.320)
* r*	Reference	0.898	0.925	0.883	0.891	0.898	0.800
TDI (95%CI), mL/min/1.73 m^2^	Reference	35.6 (27.5, 37.4)	12.5 (9.2, 14.1)	30.0 (21.4, 34.4)	30.6 (20.6, 31.7)	17.2 (12.5, 19.6)	71.1 (69.8, 72.8)
P10 performance metric (95% CI)	Reference	10.7 (5.0, 21.5)	50.0 (37.3, 62.7)	50.0 (37.3, 62.7)	23.2 (14.1, 35.8)	55.4 (42.4, 67.6)	0 (0, 6.4)
P30 performance metric (95% CI)	Reference	66.1 (53.0, 77.1)	100.0 (93.6, 100.0)	78.6 (66.2, 87.3)	78.6 (66.2, 87.3)	96.4 (87.9, 99.0)	0 (0, 6.4)
**CKiD eGFR < 75 mL/min/1.73 m**^**2**^ (***n*** **= 114)**							
eGFR, mL/min/1.73 m^2^, mean (SD)	51.7 (14.0)	66.6 (19.8)	54.6 (19.3)	61.3 (20.1)	65.3 (21.5)	58.1 (21.1)	122.3 (23.8)
Mean difference (95% CI)	Reference	14.8 (13.2, 16.5)	3.0 (1.6, 4.3)	10.0 (8.2, 11.7)	13.7 (11.7, 15.6)	6.4 (10.0)	70.7 (16.7)
ICC (95% CI)	Reference	0.863 (0.807, 0.903)	0.904 (0.864, 0.933)	0.854 (0.795, 0.897)	0.831 (0.765, 0.881)	0.846 (0.785, 0.891)	0.633 (0.509, 0.732)
CCC (95% CI)	Reference	0.628 (0.503, 0.728)	0.890 (0.845, 0.923)	0.732 (0.633, 0.807)	0.647 (0.525, 0.742)	0.795 (0.716, 0.854)	0.084 (− 0.101, 0.264)
* r*	Reference	0.916	0.951	0.910	0.909	0.921	0.726
TDI (95%CI), mL/min/1.73 m^2^	Reference	32.7 (25.8, 37.3)	16.8 (3.8, 18.5)	28.8 (22.1, 34.3)	31.1 (29.3, 33.5)	25.1 (20.5, 28.0)	102.3 (97.5, 111.8)
P10 performance metric (95% CI)	Reference	11.4 (6.8, 18.5)	38.6 (30.2, 47.8)	33.3 (25.4, 42.4)	14.9 (9.5, 22.6)	46.5 (37.6, 55.6)	0 (0, 39.3)
P30 performance metric (95% CI)	Reference	54.4 (45.3, 63.2)	99.1 (95.2, 99.8)	78.9 (70.6, 85.4)	62.3 (53.1, 70.6)	83.3 (75.4, 89.1)	50 (18.8, 81.2)
**CKiD eGFR ≥ 75 mL/min/1.73 m**^**2**^ (***n*** **= 6)**							
eGFR, mL/min/1.73 m^2^, mean (SD)	78.6 (3.5)	109.0 (6.7)	100.2 (9.0)	104.6 (5.6)	102.7 (9.0)	110.2 (12.9)	175.9 (30.9)
Mean difference (95% CI)	Reference	30.3 (4.8)	21.6 (6.1)	26.0 (4.4)	24.1 (6.9)	31.6 (10.1)	97.2 (28.5)
ICC (95% CI)	Reference	0.594 (− 0.291, 0.931)	0.608 (− 0.270, 0.934)	0.555 (− 0.343, 0.923)	0.493 (− 0.416, 0.909)	0.428 (− 0.482, 0.894)	0.158 (− 0.677, 0.815)
CCC (95%CI)	Reference	0.035 (− 0.799, 0.823)	0.102 (− 0.774, 0.844)	0.034 (− 0.800, 0.823)	0.068 (− 0.787, 0.834)	0.065 (− 0.788, 0.833)	0.015 (− 0.807, 0.817)
* r*	Reference	0.718	0.892	0.612	0.722	0.836	0.697
TDI (95%CI), mL/min/1.73 m^2^	Reference	34.9 (32.6, 35.3)	27.8 (23.0, 27.9)	29.9 (28.2, 29.9)	31.4 (26.0, 31.5)	42.0 (34.6, 42.1)	123.9 (110.0, 124.1)
P10 performance metric (95% CI)	Reference	0 (0, 39.0)	0 (0, 39.0)	0 (0, 39.0)	0 (0, 39.0)	0 (0, 39.0)	0 (0, 39.0)
P30 performance metric (95% CI)	Reference	0 (0, 39.0)	50.0 (18.8, 81.2)	33.3 (9.7, 70.0)	50.0 (18.8, 81.2)	3.0 (56.4)	0 (0, 39.0)

*95% CI* 95% confidence interval, *eGFR* estimated glomerular filtration rate, *FAS* the full age spectrum, *EKFC* European Kidney Function Consortium, *ICC* intraclass correlation, coefficient, *CCC* concordance correlation coefficient, *r* Pearson’s correlation coefficient, *TDI* total deviation index, *SD* standard deviation, *P10 performance metric* proportion within 10% of the reference, *P30 performance metric* proportion within 30% of the reference

**Fig. 1:**
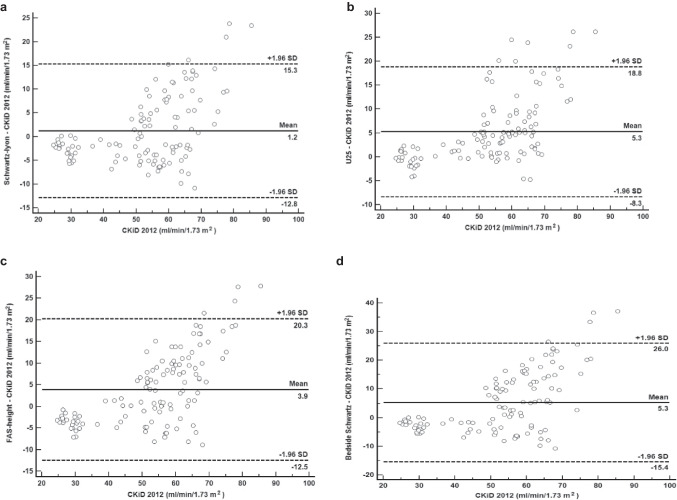
Bland‒Altman plots showing the bias and limits of agreement of PCr based and CKiD2012 combined PCr-CystC formula. The *Y*-axis represents the differences, calculated as (PCr based eGFR minus CKiD2012), for each data point. The *X*-axis represents the CKiD2012 measurements for each data point. The solid lines represent the bias; the dotted lines represent the 95% limits of agreements (mean difference ± 1.96SD). **a** Schwartz-Lyon. **b** U25. **c** FAS-height. **d** Bedside Schwartz. eGFR, estimated glomerular filtration rate; PCr, plasma creatinine; U25, CKiD under 25; FAS, full age spectrum

Bland‒Altman plots were generated to assess the bias between the CKiD 2012 combined PCr–CystC formula and four PCr-based eGFR equations: Schwartz–Lyon, U25, FAS-height, and Bedside Schwartz (Fig. 1). The plots indicated minimal bias and narrow 95% limits of agreement, suggesting low variability between these eGFR estimation methods. Additionally, Bland–Altman plots revealed proportional bias (*p* < 0.05) for all equations, prompting ROC analysis to determine clinically relevant agreement thresholds. The ROC analysis revealed that eGFR values < 70 mL/min/1.73 m^2^ for Schwartz–Lyon, U25, and FAS-height and < 60 mL/min/1.73 m^2^ for Bedside Schwartz were optimal thresholds where agreement with the reference standard remained acceptable. Beyond these thresholds, both equations showed increased bias, indicating reduced reliability in higher eGFR ranges.

### Correlation between the CKiD 2012 and U25 combined PCr-CysC equations

The U25 combined PCr-CystC equation demonstrated excellent agreement with the reference CKiD 2012 combined equation (Table 4). The mean eGFR values were similar (54.0 vs. 53.0 mL/min/1.73 m^2^), with a minimal mean difference of 1.0 mL/min/1.73 m^2^ (95% CI, 0.7, 1.3). The agreement between the two equations was very high, with an ICC of 0.993 (95% CI, 0.990, 0.995), CCC of 0.990 (95% CI, 0.969, 1.0), and a TDI of 3.9 mL/min/1.73 m^2^ (95% CI, 3.2, 5.2). The Pearson’s correlation coefficient was 0.993. Regarding performance metric, the U25 equation showed excellent performance with a P10 of 99.2% and a P30 of 100%.

**Table 4:** Correlation between the CKiD 2012 and the U25 plasma creatinine-cystatin C equations (*n* = 120)

Measurements	CKiD 2012 combined plasma creatitine-cystatin C	U25 combined plasma creatinine-cystatin C
eGFR, mL/min/1.73 m^2^, mean (SD)	53.0 (14.9)	54.0 (15.1)
Mean difference (95% CI)	Reference	1.0 (0.7, 1.3)
ICC (95% CI)	Reference	0.993 (0.990, 0.995)
CCC (95% CI)	Reference	0.990 (0.969, 1.0)
TDI (95% CI), mL/min/1.73 m^2^	Reference	3.9 (3.2, 5.2)
*r*	Reference	0.993
P10 performance metric (95% CI)	Reference	99.2 (95.4, 99.9)
P30 performance metric (95% CI)	Reference	100 (1.0, 1.0)

*95% CI* 95% confidence interval, *eGFR* estimated glomerular filtration rate, *CKiD* Chronic Kidney Disease in Children, *U25* the CKiD under 25, *CKD-EPI* Chronic Kidney Disease Epidemiology Collaboration, *MDRD* Modification of Diet in Renal Disease, *ICC* intraclass correlation coefficient, *CCC* concordance correlation coefficient, *r* Pearson’s correlation coefficient, *TDI* total deviation index, *SD* standard deviation, *P10 performance metric* proportion within 10% of the reference, *P30 performance metric* proportion within 30% of the reference

## Discussion

This study comprehensively evaluated the performance of 12 PCr-based eGFR equations in pediatric kidney transplant recipients, using the CKiD 2012 combined PCr–CystC equation as the reference standard. Among the tested equations, the Schwartz–Lyon formula showed the highest overall performance, with excellent ICC (0.913), CCC (0.911), TDI (14.0 mL/min/1.73 m^2^), and the highest P30 performance metric (99.2%). Its reliability was particularly notable in patients with eGFR < 70 mL/min/1.73 m^2^ and those aged 10–18 years. The U25, FAS–height, and Bedside Schwartz equations demonstrated comparable performance and serve as reasonable alternatives.

Recent guidelines for renal function monitoring in solid organ transplant recipients highlight the importance of selecting an appropriate GFR estimation method tailored to the patient’s physiological characteristics [17]. The KDIGO 2024 guidelines reinforce the role of the combined PCr-CystC-based formula in clinical decision-making, particularly when the PCr-based formula alone is less reliable [17]. Pizzo et al. assessed mGFR in 45 pediatric kidney transplant recipients using several equations, including the CKiD 2012 and U25 combined PCr–CystC formulas, and found both performed similarly and ranked among the best estimators [31]. In our study, which used the CKiD 2012 combined equation as the reference, the U25 combined equation also demonstrated excellent agreement (ICC = 0.993, CCC = 0.990, TDI = 3.9 mL/min/1.73 m^2^, P30 = 100%), supporting its potential as an alternative equation when CystC is available.

Our study showed that the Schwartz–Lyon equation had the best overall performance, particularly in patients with eGFR < 70 mL/min/1.73 m^2^ and those aged 10–18 years. The U25, FAS–height, and Bedside Schwartz also performed comparably and support previous findings on the value of pediatric-specific formulas for estimating GFR in this population [7, 9, 10, 19]. Compared with previous pediatric studies, the findings align closely with those of de Souza et al. [7], who reported that the Schwartz–Lyon equation demonstrated good performance with respect to mGFR, particularly at lower GFR levels (mGFR < 60 mL/min/1.73 m^2^), with high P30 accuracy rates (97–98%) and better reliability than other PCr-based equations. Additionally, Papez et al. evaluated eGFR estimation in a Hispanic-dominant pediatric kidney transplant population and reported that the Bedside Schwartz equation demonstrated the highest correlations with mGFR using iothalamate clearance [9]. A previous study by Tsampalieros et al. compared the Bedside Schwartz eGFR with the CKiD 2009 combined with the PCr-CysC formula in pediatric kidney transplant recipients and reported that the Bedside Schwartz formula overestimated the GFR, with a mean bias of 1.09 mL/min/1.73 m^2^ [32]. However, they did not include the Schwartz–Lyon equation. A previous Canadian study by Alkandari et al. found that the modified Schwartz and FAS-age equations demonstrated better bias and P30 accuracy in pediatric kidney transplant recipients [10], although FAS-height was not assessed. In contrast, our study demonstrated superior performance of FAS-height over FAS-age. This discrepancy may reflect population-specific growth patterns. In our Thai cohort, post-transplant growth retardation is common, making age a less reliable proxy for body size. As FAS-height incorporates actual stature, it may better capture renal function in this population. Conversely, the Canadian cohort may have had growth patterns more aligned with the original European derivation cohort of FAS-age, contributing to its better performance in that setting.

A previous study conducted by de Souza et al. analyzed the performance of different eGFR equations in pediatric kidney transplant recipients and assessed their ability to correctly classify renal dysfunction using AUC analysis. They reported that the Schwartz–Lyon and Bedside Schwartz AUCs were 0.97 and 0.96, respectively, for detecting an mGFR < 60 mL/min/1.73 m^2^ [7]. Consistent with our findings, this study reinforces the need for equation-specific eGFR thresholds, as identified by our ROC analysis (< 70 mL/min/1.73 m^2^ for Schwartz–Lyon, U25, and FAS-height and < 60 mL/min/1.73 m^2^ or Bedside Schwartz). Additionally, a Canadian study by Alkandari et al. found that the modified Schwartz performed best in children aged 5–15 years. In our study, the Bedside Schwartz showed better performance in those aged 10–18 years. T﻿hese results highlight the importance of selecting eGFR equations with high classification accuracy, particularly in populations at risk of developing allograft dysfunction.

This study has several strengths. First, our study comprehensively compared several PCr-based eGFR equations. This comprehensive comparison provides substantial advantages over studies that evaluate only a limited number of equations. Second, this study focused on a unique clinical population of pediatric kidney transplant recipients receiving adult donor kidneys. This focus provides valuable insights into the performance of eGFR equations tailored to the unique physiological and clinical characteristics of these patients, thereby filling a critical gap in nephrology research. Third, the application of advanced statistical analyses, including Bland‒Altman plots, ICCs, and performance metrics such as P10 and P30 performance metrics, ensures a thorough assessment of each eGFR formula’s reliability and agreement with the reference standard. These methods are widely recognized for their effectiveness in method comparison studies, enhancing the credibility of the results. Fourth, ROC analysis was used to define eGFR thresholds at which each equation maintains optimal accuracy, and subgroup analyses by age were performed. This approach enhances clinical applicability by offering more precise guidance for renal function assessment in pediatric kidney transplant recipients.

There are some limitations in this study. First, although the sample size calculation indicated the need for at least 120 samples to demonstrate a significant difference [7], this study included only 23 patients because of the limited number of transplant recipients at our center during the study period. However, each individual measurement remained independent and did not exert any influence on the others. Second, there is a lack of direct mGFR assessments to confirm the absolute accuracy of the eGFR equations. While eGFR equations are widely used and practical for clinical application, they may not always precisely reflect the true GFR. Consequently, our study relies on comparative analyses between different eGFR equations rather than direct GFR measurements. Third, while corticosteroids may affect CystC levels, a prior study in pediatric kidney transplant recipients revealed no significant correlation between CystC levels and prednisolone use [33]. Furthermore, this study included only patients receiving low-dose prednisolone (< 0.5 mg/kg/day), which may have minimized any potential impact on CystC levels. Fourth, although timing post-transplant may influence the performance of eGFR equations, our dataset included only four eGFR measurements obtained within the first 3 months after transplantation. Due to this limited sample size, we were unable to perform a dedicated subgroup analysis for this early post-kidney transplant period. Fifth, the CKiD 2012 equation was developed in children with mostly stage 3 or higher CKD and may not fully reflect transplant recipients with near-normal GFR [13]. In our study, most equations performed better in the < 75 mL/min/1.73 m^2^ group. Similarly, a recent study noted reduced accuracy of eGFR equations at higher GFR levels, highlighting the need for improved models for children with preserved kidney function [34]. Future research should involve larger, multicenter studies—including patients in the early post-kidney transplant period and incorporating direct GFR measurements to increase the validity and reliability of kidney function assessment methods in pediatric kidney transplant recipients.

In conclusion, the Schwartz–Lyon equation demonstrated the highest performance in estimating the eGFR in pediatric kidney transplant recipients, particularly when eGFR was < 70 mL/min/1.73 m^2^ and in patients aged 10–18 years, and should be considered the preferred equation in this population when CystC is not available. Notably, the U25 combined PCr-CystC equation showed the best overall agreement of the reference CKiD 2012 combined equation and may be preferred in clinical settings when CystC measurement is feasible. The U25, FAS–height, and Bedside Schwartz equations demonstrated comparable performance and serve as reasonable alternatives. These findings reinforce the importance of selecting appropriate eGFR estimation methods tailored to the physiology of pediatric kidney transplant patients.

## Supplementary information

Below is the link to the electronic supplementary material.

Graphical abstract (PPTX 258 KB)
