# Supplementary material for: Comparison of different equations for estimating the glomerular filtration rate in pediatric kidney transplant recipients
Source: Pediatr Nephrol. 2025 Sep 22;41(1):203–16. doi: 10.1007/s00467-025-06942-8 (PMC12686102; doi:10.1007/s00467-025-06942-8)
Supplement: Supplementary file 1 — Graphical abstract (PPTX 258 KB) [file 467_2025_6942_MOESM1_ESM.pptx]

## Slide 1
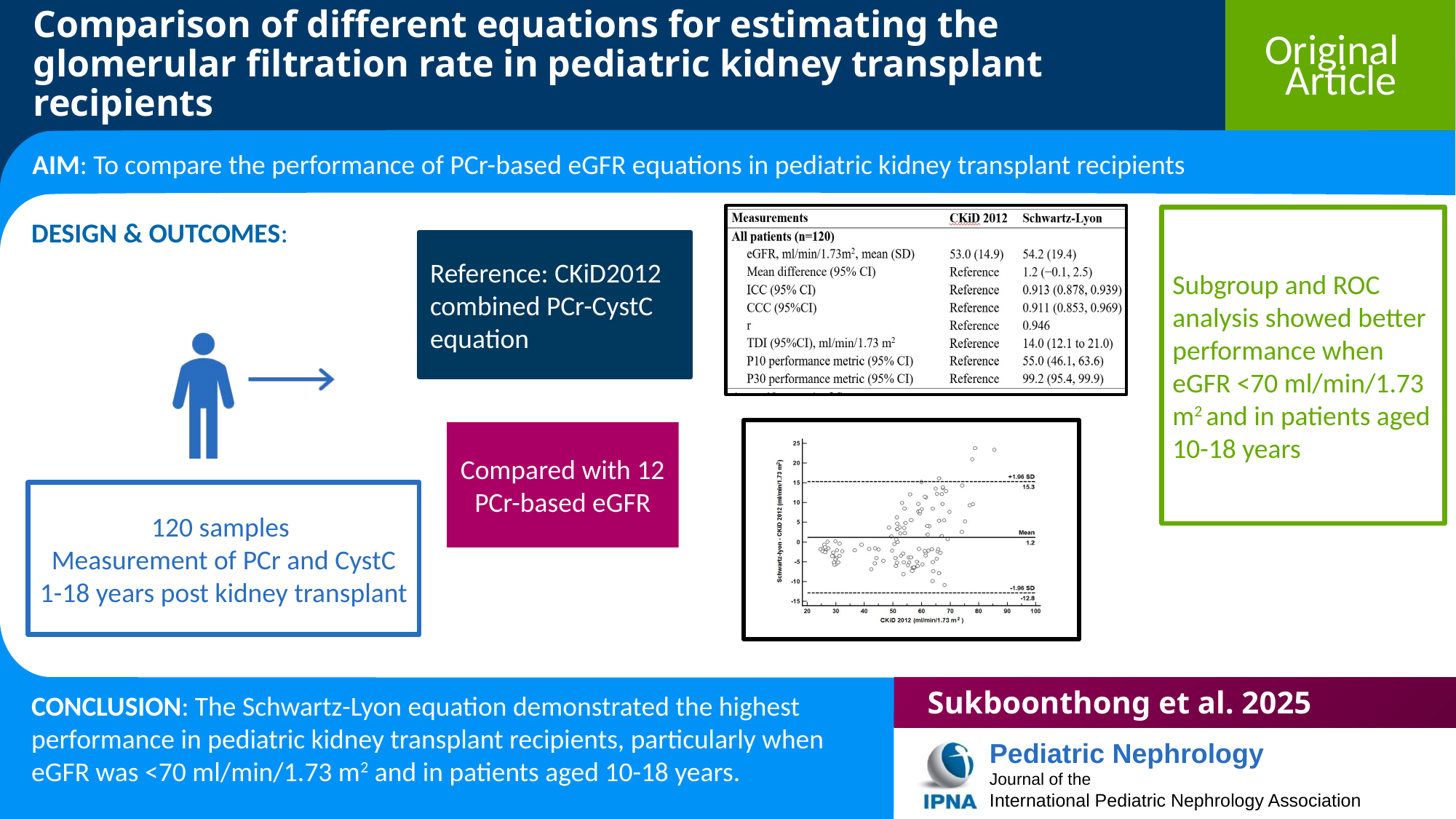

Comparison of different equations for estimating the glomerular filtration rate in pediatric kidney transplant recipients
AIM: To compare the performance of PCr-based eGFR equations in pediatric kidney transplant recipients
Subgroup and ROC analysis showed better performance when eGFR <70 ml/min/1.73 m2 and in patients aged 10-18 years
DESIGN & OUTCOMES:
Reference: CKiD2012 combined PCr-CystC equation
Compared with 12 PCr-based eGFR
120 samples
Measurement of PCr and CystC
1-18 years post kidney transplant
Sukboonthong et al. 2025
CONCLUSION: The Schwartz-Lyon equation demonstrated the highest performance in pediatric kidney transplant recipients, particularly when eGFR was <70 ml/min/1.73 m2 and in patients aged 10-18 years.
